# Dietary patterns, obesity markers and leukocyte telomere length among Brazilian civil servants: cross-sectional results from the Pro-Saude study

**DOI:** 10.1017/S1368980023001064

**Published:** 2023-10

**Authors:** Nathalia Ferrazzo Naspolini, Rosely Sichieri, Diana Barbosa Cunha, Rosangela Alves Pereira, Eduardo Faerstein

**Affiliations:** 1 Instituto de Medicina Social, Universidade do Estado do Rio de Janeiro, Rio de Janeiro, RJ 20550-900, Brasil; 2 Universidade Federal do Rio de Janeiro, Departamento de Nutrição Social e Aplicada, Rio de Janeiro, RJ, Brasil

**Keywords:** Dietary patterns, Partial least squares, Obesity, Telomere length

## Abstract

**Objective:**

Dietary patterns express the combination and variety of foods in the diet. The partial least squares method allows extracting dietary patterns related to a specific health outcome. Few studies have evaluated obesity-related dietary patterns associated with telomeres length. This study aims to identify dietary patterns explaining obesity markers and to assess their association with leukocyte telomere length (LTL), a biological marker of the ageing process.

**Design:**

Cross-sectional study.

**Setting:**

University campuses in the state of Rio de Janeiro, Brazil

**Participants:**

478 participants of a civil servants’ cohort study with data on food consumption, obesity measurements (total body fat, visceral fat, BMI, leptin and adiponectin) and blood samples.

**Results:**

Three dietary patterns were extracted: (1) fast food and meat; (2) healthy and (3) traditional pattern, which included rice and beans, the staple foods most consumed in Brazil. All three dietary patterns explained 23·2 % of food consumption variation and 10·7 % of the obesity-related variables. The fast food and meat pattern were the first factor extracted, explaining 11–13 % variation of the obesity-related response variables (BMI, total body fat and visceral fat), leptin and adiponectin showed the lowest percentage (4·5–0·1 %). The healthy pattern mostly explained leptin and adiponectin variations (10·7 and 3·3 %, respectively). The traditional pattern was associated with LTL (*β* = 0·0117; 95 % CI 0·0001, 0·0233) after adjustment for the other patterns, age, sex, exercise practice, income and energy intake.

**Conclusion:**

Leukocyte telomere length was longer among participants eating a traditional dietary pattern that combines fruit, vegetables and beans.

Over the past four decades, the global prevalence of obesity has nearly tripled^(1)^, making it a global pandemic^(2)^. In Brazil, the prevalence of obesity in adults increased by 54 % from 2003 to 2019 and the prevalence of overweight increased by 30 % in the same period^(3)^. Obesity increases the risk of many non-communicable chronic diseases^(4)^. In 2016, an estimated 5 million deaths were attributed to overweight and obesity worldwide^(5)^.

The mechanisms linking obesity to many non-communicable chronic diseases encompass dysfunctional adipose tissue inducing systemic low-grade inflammation and oxidative stress^(6,7)^. The increased levels of proinflammatory cytokines and acute-phase proteins are related to the secretory capacity of the adipose tissue and its dysfunctional characteristic in this condition. Systemic inflammation linked to obesity seems to be determinant for the development of several comorbidities, such as insulin resistance, type 2 diabetes mellitus and CVD^(8)^. Telomere length may be influenced by obesity-related inflammation and oxidative stress^(9)^.

Telomeres are regions of repeated nucleotide sequences (TTAGGG in humans), situated at both ends of each chromosome and protect against chromosome degradation and inter-chromosomal fusion. Telomeres become gradually shorter with increasing age due to DNA loss during cell division, representing a biologic marker of ageing^(10)^. A meta-analysis of eighty-seven observational studies found an inverse association between BMI and telomere length^(11)^. Yet, no prior studies have focussed on the impact of diet and dietary patterns associated with obesity that may modify the telomeres. The most consistent association found by reviews was with Mediterranean diet, with higher adherence to this dietary pattern associated with longer telomeres^(12,13)^.

Exploratory dietary pattern analyses could realistically reflect dietary habits by examining the effects of the total diet. The partial least squares (PLS) method combines an exploratory analysis, with a priori hypothesis on the role of intermediate variables on the outcome. This hybrid methodologies have gained interest as unique data reduction techniques for establishing a direct link between dietary exposures and health outcomes^(14)^.

Nevertheless, studies evaluating dietary patterns explaining obesity-related markers and their association with telomeres length have been poorly described. In the present study, we used the PLS method to identify dietary patterns explaining the following obesity-related markers: body fat, visceral fat, BMI, leptin, adiponectin and to further assess their association with leukocyte telomere length (LTL).

## Methods

### Study design and population

A cross-sectional analysis was conducted on the Pró-Saúde Study, a civil servant-based cohort study at university campuses in the state of Rio de Janeiro, Brazil. Briefly, four waves of data collection were conducted among 3253 participants (1999, 2001, 2006, 2012). During wave four (2012), a random sample of 520 participants stratified by sex, age (< 50 *v*. > 50 years) and education level (< high school *v*. > high school) from the Pró-Saúde baseline population was selected to perform additional interview, anthropometric and body composition assessment and collection of blood samples. Details on the study design and cohort profile can be found elsewhere^(15)^. Individuals with missing data for obesity measurements and diet (*n* 42) were excluded. Therefore, the current analysis includes data from 478 participants.

All field workers were trained, and all measurements underwent strict quality control with respect to the data collection process. In parallel with data collection, questionnaires were evaluated and coded by field supervisors, independently entered by two research assistants and stored in EpiData 3·1 (EpiData Association, Odense, Denmark). Reporting of clinical data complies to the STROBE guidelines.

### Diet assessment

Food consumption was assessed by using a self-reported semi-quantitative FFQ, validated by Sichieri et al.^(16)^, structured on eighty-two food items or food groups. Food amounts are predefined and expressed as household measures or food units. Respondents selected from eight consumption frequencies (> 3 times/d; 2–3 times/d; 1 time/d; 5–6 times/week; 2–4 times/week; 1 time/week; 1–3 times month; never or almost never), converted into grams per day. The nutrient and energy contents of the foods was derived from the Brazilian Table of Food Compositions^(17)^. The usual portion/serving size was used to estimate the daily consumption. The eighty-two-item FFQ were assigned into twenty-three food groups (online Supplementary Table 1) according to composition. The total dietary energy was calculated, and to reduce spurious variability, the dietary energy density was estimated by dividing food amounts (grams) by the daily energy consumed (kJ)^(18)^.

### Adiposity assessment

The total body mass, total body fat and visceral fat were measured via DXA with iDXA Lunar equipment (GE Healthcare, WI) and enCORE 2008 version 12.20 software^(19)^. For the full-body examination, participants wore light clothing and put-off metal accessories. Participants were placed in the dorsal recumbent position and asked to remain motionless until the end of the procedure. All scans were performed by the same trained professional, and the equipment was calibrated daily according to the manufacturer’s protocol. Measurements of the manufacturer-supplied calibration block (daily) and column calibration block (weekly) showed a < 0·7 % CV^(19)^. The total body fat was expressed in kg and as a percentage. Visceral fat was expressed as a percentage of the total fat mass obtained via DXA.

Body mass (kg) and height (m) were measured by trained research assistants, using a digital scale with accuracy of 0·1 kg and a fixed stadiometer (SECA) with accuracy of 0·1 cm. BMI (BMI = body mass/height^2^) of each participant was calculated (kg/m^2^).

### Blood collection and biochemical biomarkers assessment

Blood samples were collected after 12-h overnight fast by a trained professional, using vacutainer tubes (Becton, Dickinson & Company). Serum samples were collected and stored at –80°C until analyses. Plasma leptin (Cat. #ZHL-80SK) and adiponectin (Cat. #EZHADP-61K) levels were determined using semiautomated chemiluminescent enzyme-labelled immunometric assay (Liaison). Intra-assay coefficients of variation were < 6 %.

### Leukocyte telomere length assessment (LTL)

DNA samples were isolated from whole blood using a commercial kit (Puregene Blood Kit Qiagen). The quality and concentration of DNA samples were evaluated by spectrophotometry (BioDrop DUO, BioDrop) and were stored at –80°C, until the determination of LTL and genotyping. LTL was measured by quantitative real-time PCR (qPCR) based on the method described by Cawthon with modifications^(20,21)^. Reactions were conducted in triplicate and included genomic DNA (1·6 ng), 2 × RotorGene SYBR Green, PCR Master Mix (Qiagen), primers Tel Forward (300 nM -CGGTTTGTTTGGGTTTGGGTTTGGGTTTGGGTTTGGGTT) and Tel Reverse (300 nM -GGCTTGCCTTACCCTTACCCTTACCCTTACCCTTACCCT) or primer single gene Hbg1 Forward (300 nM -GCTTCTGACACAACTGTGTTCACTAGC) and single gene Hbg2 Reverse (700 nM -CACCAACTTCATCCACGTTCACC) (Integrated DNATechnologies), in a 24 µl reaction. Amplification was conducted in a Rotor-Gene Q Real-Time PCR cycler (Qiagen) as follows: 95°C by 5 min, 25 cycles (telomere reaction) or 35 cycles (single gene reaction) at 98°C by 7s and 60°C by 10 s. The telomere length for each sample was determined using the telomere-to-single copy gene ratio (T/S ratio) by the ΔCt method (Ct[telomere]/Ct [single gene]). The T/S ratio for each sample (x) was normalised to the mean a T/S ratio of reference sample (r) [2 (ΔCtx -ΔCtr)=2-ΔΔCt], which was also used for the standard curve, both as a reference sample and as an interassay validation sample. All sample data included had reference samples with an interassay CV < 9 %. For qPCR validation purposes, the LTL was evaluated in a random sample (10 %) using the Southern Blot method of terminal restriction fragment, as described by Gutierrez-Rodrigues et al.^(22)^. The analysis was conducted using a commercial kit (Telo TAGGG Telomere Length Assay Roche Applied Science).

### Covariates

Information on socio-demographic variables was collected using a self-report questionnaire. Variables considered in this study were sex (male/female), age, *per capta* family income, and energy intake (kJ/d) and exercise practice. The last was assessed by means of a yes/no question: ‘In the last 2 weeks, did you engage in any physical activity to improve your health and physical condition or for fitness or leisure purposes?’.

### Statistical analyses

Characteristics of participants were described as means and standard deviations, for continuous variables or as proportions (%) for categorical variables. PLS method was used to derive dietary patterns that explained maximum variation in obesity-related response variables: total body fat (kg), visceral fat (kg), BMI (kg/m^2^), leptin (ng/ml) and adiponectin (ng/ml). The response variables used in this study were chosen a priori and used as proxy for obesity and mediators of the association between dietary patterns and LTL^(11,23)^.

Based on the cumulative percentage of explained variance and interpretability of the dietary patterns, three factors were set to be retained in the models. Food groups having factor loadings > |0·15| were considered relevant in the definition of the dietary patterns. Subsequent regression analyses were performed to estimate the association between the dietary patterns factors scores and LTL. Potential confounding effects of age, sex, exercise practice, income and energy intake were adjusted in the model. The significance level for all analysis was set at 5 %. All analyses were conducted in SAS (SAS Institute Inc.).

## Results

The sub-sample of 478 participants from the Pró-Saúde Study does not differ at the baseline from the overall cohort in relation to age, exercise practice and obesity. Individuals aged 33–79 years (mean: 51·6 ± 7·9 years), with 52·3 % women. Prevalence of obesity was 32·5 % and about 40 % were physically active (Table 1).

**Table 1:** Sample size, means, percentages and sd of the study population. Pro-Saúde Study—Rio de Janeiro, Brazil, 2012–2013

	*n*	Mean	sd
Age, years	478	51·7	7·7
BMI (kg/m^2)^	478	28·1	5·1
Height (cm)	478	165·9	9·2
Total body lean mass (kg)	478	46·1	9·8
Total body fat (kg)	478	28·1	9·9
Visceral fat (kg)	478	13·2	8·9
Energy intake, kJ/d	478	10406·4	3858·1
Leukocyte telomere length, db	478	0·58	0·15
Leptin	478	6·3	5·7
Adiponectin	478	53·4	46·5

	*n*	%	
Sex			
Male	228	47·7	
Female	250	52·3	
BMI categories*			
Underweight	4	0·8	
Healthy weight	132	27·6	
Overweight	188	39·3	
Obesity	154	32·3	
Physically active†	194	40·6	

* BMI < 18·5 kg/m^2^: underweight; 18·5–24·9 kg/m^2^: healthy weight; 25–29·9 kg/m^2^: overweight; > 30 kg/m^2^: obesity.

† Any exercise practice in the last 2 weeks.

Factor loadings of the first three dietary patterns derived by PLS method are shown in Table 2. Food groups with absolute values of loadings ≥ 0·15 are highlighted. The first pattern was called ‘fast food and meat’, consisting of soft drinks, cold cuts, fast foods and savoury snacks, alcoholic beverages and red meat as positive contributors while sugar, beans, roots, pulses, milk and dairy, cake and pastries were inverse contributors. The second pattern, called ‘healthy’ pattern had positive loadings for milk and dairy, fresh fruits, vegetables and greens, caffeinated drinks, pulses, fruit juices and fish and negative loadings on beans, rice, pasta, alcoholic beverages, red meat and soft drinks. The ‘traditional’ pattern had positive loadings for usually consumed staple foods such as rice, beans, vegetables and greens. Fruits, fish and fruit juices were also included in this pattern, while negative loadings were estimated for cake and pastries and fast food and savoury snacks (Table 2).

**Table 2:** Food items and groups, model effect loadings and explained variance (%) from dietary patterns associated to obesity derived from partial least squares (PLS)

	Model effect loadings		
Food items/groups	1 ‘Fast food and Meat’	2 ‘Healthy’	3 ‘Traditional’
Soft drinks	**0·435**	**−0·177**	−0·110
Cold cuts	**0·406**	−0·030	−0·111
Sugar	**−0·340**	−0·125	−0·141
Beans	**−0·318**	**−0·348**	**0·277**
Fast foods, savoury snacks	**0·303**	−0·088	**−0·329**
Roots	**−0·232**	−0·077	0·009
Alcoholic beverages	**0·213**	**−0·244**	0·088
Red meat	**0·209**	**−0·209**	−0·117
Pulses	**−0·199**	**0·179**	0·092
Milk and dairy	**−0·197**	**0·356**	0·082
Cake and pastries	**−0·195**	0·098	**−0·41**
Rice	−0·105	**−0·343**	**0·409**
Fresh fruits	−0·101	**0·348**	**0·282**
Fish	0·100	**0·168**	**0·256**
Caffeinated drinks	0·064	**0·213**	0·117
Vegetables and greens	0·039	**0·326**	**0·391**
Pasta	−0·017	**−0·280**	−0·073
Fruit juices	0·016	**0·169**	**0·224**
Cumulate total explained variation (%)	6·71	15·17	23·22

Response variables	% variation accounted for		
Leptin	4·55	10·67	10·68
Adiponectin	0·06	3·27	4·69
Total body fat (kg)	13·04	13·79	14·13
Visceral fat (kg)	7·11	11·18	11·60
BMI	11·40	11·41	12·35
Cumulate total explained variation (%)	7·23	10·07	10·69

For food items/groups values are loadings and for response variables values are % variation accounted for by PLS factors.

Remaining food groups loadings had < |0·15|: fats (butter and mayonnaise), bread, eggs, healthy snacks (peanuts and popcorn), meats (pork, chicken and tripe); Number of participants included in the model = 478.

The three dietary patterns together explained 23·2 % of the variation of the predictive variables (food groups/items) and 10·7 % of the response variables variance. Among the response variables, 14·1 % of total body fat is explained by the variation in food consumption. The other anthropometric measures had similar percentage values (12·3 % for BMI and 11·6 % for visceral fat), whereas adiponectin showed the lowest percentage (4·7 %).

The fast food and meat dietary pattern explained 13 % of total body fat, 7·1 % of visceral fat and 11·4 % of BMI variations, explaining 7·2 % of the response variables in total. It also explained 4·5 % of leptin and 0·1 % of adiponectin variations. The healthy pattern mostly explained leptin and adiponectin variations (10·7 and 3·3 %, respectively) (Table 2).

The traditional dietary pattern was directly associated with LTL (*β* = 0·0117; 95 % CI 0·0001, 0·0233) in a model including all three patterns and adjusted for age, sex, physical activity, income and energy intake (Table 3). Separate models for each pattern showed similar results (Table 3).

**Table 3:** Regression coefficients between the identified dietary patterns and telomere length. Pro-Saúde Study—Rio de Janeiro, Brazil, 2012–2013

	Crude *β*	95 % CI	Adjusted* *β*	95 % CI	Adjusted† *β*	95 % CI
**Models including all three dietary patterns**						
Fast food and Meat	0·0069	−0·0043, 0·0181	0·0016	−0·0096, 0·0128	0·0067	−0·0153, 0·0287
Healthy	−0·0095	−0·0196, 0·0066	−0·0108	−0·0243, 0·0027	−0·0086	−0·0268, 0·0096
Traditional	0·0085	−0·0017, 0·0187	**0·0107**	**0·0003, 0·0211**	**0·0117**	**0·0001, 0·0233**
**Models for each pattern**						
Fast food and Meat	0·0024	−0·0060, 0·0108	−0·0027	−0·0121, 0·0067	−0·0045	−0·0167, 0·0077
Healthy	−0·0043	−0·0143, 0·005	−0·0101	−0·0213, 0·0011	−0·0046	−0·0148, 0·0056
Traditional	0·0094	−0·0008, 0·0196	**0·0112**	**0·0010, 0·0214**	**0·0109**	**0·0001, 0·0219**

* Adjusted for age, sex and exercise practice.

† Adjusted for age, sex. exercise practice and energy intake.

## Discussion

The present study identified three dietary patterns related to obesity markers: ‘fast food and meat’, ‘healthy’ and ‘traditional’. The fast food and meat pattern explained most of the variation of the obesity-related response variables and the traditional pattern was directly associated with LTL independent of sex, age, exercise practice, income and energy intake.

Fast foods are energy-dense, high-fat and low-fibre content and the consumption of these foods has been associated with obesity worldwide^(24–26)^. This pattern of diet is usually identified as western, and in a study in Iran, it was directly associated with BMI and fat mass, whereas traditional pattern was inversely associated with dietary inflammatory index and insulin resistance among healthy adults with obesity^(27)^. This corroborates our findings, showing that the major dietary pattern responsible for explaining the obesity-related variables was the fast food and meat pattern, responsible for 7 % of the response variables variance.

In contrast, healthy patterns have been inversely associated with BMI, waist circumference and other obesity-related biomarkers^(25,28)^. In Brazil, the traditional dietary pattern, which is considered a healthy one and is heavily based on the consumption of rice and beans. This pattern was protective for obesity in studies with adults^(29,30)^ and adolescents^(31,32)^. Authors have attributed this protective effect to the high fibre content of rice and beans combination resulting in low glycaemic index meals^(30)^. Besides, the consumption of these foods seems to be a marker of home-cooked meals that are more likely to contain vegetables and whole grains^(33)^. Also, the consumption of black bean, the most consumed bean in Rio de Janeiro, is associated with reduced percentage of body fat, serum leptin, glucose and insulin concentrations, results that could be mediated in part by modification of the gut microbiota^(34)^.

Regarding the adipokines evaluated in this study, leptin and adiponectin have important role in regulating inflammation, especially in obesity^(8)^. We found that the healthy and traditional patterns were responsible for explaining the variation of leptin (10·7 % for both). Leptin is primarily known as a satiety hormone and a pro-inflammatory adipokine, its serum levels and gene expression in adipocytes strongly correlate with the proportion of body fat stores^(8)^. The close values for the explanation of leptin by the healthy and traditional patterns may be due to the high prevalence of overweight and obesity in this population. On the other hand, adiponectin’s variation was mostly explained (4·7 %) by the traditional pattern. Adiponectin is an anti-inflammatory factor and its production is frequently reduced in obesity^(35)^. However, in other studies, adiponectin’s levels are mostly correlated to ‘Western-type’ and meat-based patterns and ultra-processed food consumption^(36,37)^, and the later association was mediated by the presence of obesity. This divergence could be related to the dual activity of adiponectin, which acts as an anti-inflammatory factor especially in atherosclerosis, but in some chronic inflammatory/autoimmune diseases adiponectin may have pro-inflammatory effects^(8)^. Even being responsible for explaining, in part, the variation of these adipokines, the traditional patterns did not add up to the explanation of the obesity-related variables.

As observed in many countries, including Brazil^(38)^ there is a trend of reduction in the consumption of traditional foods and substitution by ultra-processed foods. High intake of ultra-processed foods has been associated with a higher risk of having shorter telomeres in an elderly Spanish population^(39)^. In contrast, the traditional dietary pattern in Costa Rica – which also has rice and beans as staple food – was directly associated with telomere length in an elderly population^(40)^. A systematic review and meta-analysis of eight cross-sectional studies demonstrated that higher Mediterranean diet adherence is associated with longer telomere^(12)^. The Mediterranean diet is a traditional dietary patter in countries such as Greece, Spain and Italy in the period of pre-globalisation of the food system. Another narrative review of Mediterranean diet demonstrated its positive influence on longevity^(13)^. Moreover, telomere length was linearly related to fruits and vegetables intake in a study of 5448 U.S. adults^(41)^.

The traditional dietary pattern identified in our analysis has common characteristics with the Mediterranean dietary pattern, such as high consumption of vegetables including green vegetables, fruits and beans (legumes/pulses) and, moderate intakes of fish, and low intakes of sweets; and differs regarding the consumption of extra virgin olive oil, nuts, dairy products, and red wine^(42)^ – which are not usually consumed. Beyond other positive aspects of a traditional food intake, such as reduction in weight gain, our results indicate a role in longevity reinforcing dietary guidelines of increasing intake of the traditional Brazilian dietary pattern. Together with the positive findings in other countries, our results consolidate a protective role for traditional dietary patterns.

The limitation of the study is the cross-sectional analysis of the cohort since the LTL measure and food consumption was done after 13 years of follow-up. This may explain the similar estimate values, although in opposite direction, for healthy and traditional patterns. It might represent a higher adherence to the healthy pattern among individuals with higher BMI since reverse causality cannot be ruled out. Furthermore, our study sample of civil servants limits the possibility to extrapolate results to the general population. However, we showed that the subsample with LTL measures had similar distribution by age, BMI and exercise practice compared to the baseline.

This study also has strengths, particularly the use of the PLS to identify eating patterns. The PLS is a hybrid method that extracts a latent factor simultaneously related to a set of independent variables and to a set of dependent variables, in such way that the explanation of the covariance between the two set of variables is maximised and a direct link between dietary exposures and clinical outcomes is established. In this case, data reduction of food groups is directed by biomarkers of interest. Therefore, this technique may be more suitable to extract dietary patterns when the objective is to explain the effect of intermediary variables in the relationship of eating patterns and an outcome.

Our results indicate that the consumption of traditional and culturally acceptable foods must be encouraged to promote health, longevity and control obesity.

## Supplementary material

For supplementary material accompanying this paper visit https://doi.org/10.1017/S1368980023001064. click here to view supplementary material
