# Dietary patterns, obesity markers and leukocyte telomere length among Brazilian civil servants: cross-sectional results from the Pro-Saude study – CORRIGENDUM

**DOI:** 10.1017/S1368980023001477

**Published:** 2023-10

**Authors:** Nathalia Ferrazzo Naspolini, Rosely Sichieri, Diana Barbosa Cunha, Rosangela Alves Pereira, Eduardo Faerstein

A previous version of this article had Diana Barbosa Cunha’s name in the wrong order. The article has since been updated to display the author’s correct name.
