# Supplementary material for: Dietary patterns, obesity markers and leukocyte telomere length among Brazilian civil servants: cross-sectional results from the Pro-Saude study
Source: Public Health Nutr. 2023 May 26;26(10):2076–82. doi: 10.1017/S1368980023001064 (PMC10564599; doi:10.1017/S1368980023001064)
Supplement: Supplementary file 1 [file S1368980023001064sup001.docx]

**Supplementary Material**

Suppl. Table 1. Food groups composition and food items from the FFQ. Pro-Saúde Study—Rio de Janeiro, Brazil, 2012–2013.

| Groups | Foods |
| --- | --- |
| Soft drinks | Soft drinks |
| Processed meats | Sausages, ham, bacon, hamburger |
| Sugar | Sugar |
| Beans | Black beans |
| Fast foods and savory snacks | Fried chips, other salty snacks, salted biscuits and pizza |
| Roots | Manioc, cassava flour, yams and baked potatoes |
| Alcoholic beverages | Bear, wine and liquors |
| Red meat | Beef and barbecue |
| Pulses | Lentils, dry peas and chickpeas |
| Milk and dairy | Milk, yogurt, cheese and cream cheese |
| Cake and pastries | Cakes, ice cream, cadies, sweated fruits, sweet biscuits, chocolate (bars and powder), pudding and mousse |
| Rice | Rice |
| Fresh fruits | Orange, tangerine, banana, pears, pineapple, apples, guava, melon or watermelon, avocado, mango, passion fruit and grapes |
| Fish | Fresh fish, sardines and tuna fish |
| Caffeinated drinks | Yerba matte and coffee |
| Vegetables and greens | Chayote, okra, cucumber, beets, carrots, onion, garlic, peppers, tomatoes, zucchini, pumpkin, green beans, cauliflower, lettuce, cabbage and chicory |
| Pasta | Spaghetti and lasagna |
| Fruit juices | Fruit juices |
| Remaining food groups (loadings < \|0.15\|) |  |
| Fats | Butter and mayonnaise |
| Eggs | Eggs |
| Healthy snacks | Peanuts and popcorn |
| Bread | French bread or loaf bread |
| Meats | Pork, chicken and tripe |
